# Can the neural–cortisol association be moderated by experience-induced changes in awareness?

**DOI:** 10.1038/srep16620

**Published:** 2015-11-18

**Authors:** Way K. W. Lau, Mei-Kei Leung, Chetwyn C. H. Chan, Samuel S. Y. Wong, Tatia M. C. Lee

**Affiliations:** 1 Laboratory of Cognitive Affective Neuroscience, The University of Hong Kong, Hong Kong; 2 Laboratory of Neuropsychology, The University of Hong Kong, Hong Kong; 3 Applied Cognitive Neuroscience Laboratory, Department of Rehabilitation Sciences, The Hong Kong Polytechnic University, Hong Kong; 4 School of Public Health, The Chinese University of Hong Kong, Hong Kong; 5 The State Key Laboratory of Brain and Cognitive Sciences, The University of Hong Kong, Hong Kong; 6 Institute of Clinical Neuropsychology, The University of Hong Kong, Hong Kong

## Abstract

Cortisol homeostasis is important for cognitive and affective functions that depend on cortisol-sensitive brain regions including the hippocampus and prefrontal cortex. Recent studies have shown that training induces changes in the brain. We report the findings of a longitudinal study that verified the moderation effect of experience-induced changes in awareness on the neural–cortisol association in cortisol-sensitive brain regions. These findings provide the first piece of evidence that planned behavioral experience can moderate the neural–cortisol association. A range of changes in awareness was achieved in a sample of 21 Chinese participants, divided into two groups: Awareness-based compassion meditation (ABCM) (n = 10) and relaxation (n = 11). We observed that changes in awareness were significant moderators of hippocampal–cortisol changes. Furthermore, a significant negative association between changes in plasma cortisol level and the resting-state synchrony of the right hippocampal and insular-frontal-operculum regions was observed. These novel findings shed light on the inter-relationships between changes in hippocampal–cortisol levels and changes in awareness and preliminarily identify the neural underpinnings of interventions for cortisol-related abnormal functioning for further study.

Neuroplasticity underlies changes in neural pathways and synapses of the human neural system, and it plays an important role in the modification of neural communication with the endocrine system to produce adaptive responses to life changes^1^,^2^. In fact, the nervous and endocrine systems contribute to a large proportion of human bodily functions, and these two systems are inter-related via different pathways. One of the well-studied pathways is the hypothalamus-pituitary-adrenal (HPA) axis, which regulates peripheral cortisol levels. Disruption of cortisol homeostasis is associated with various cognitive and affective dysfunctions. For example, elevated plasma cortisol levels are associated with major depressive disorder^3^, and lower plasma cortisol levels may be associated with emotional numbing symptomatology in people with posttraumatic stress disorder^4^. Cortisol also plays a role in neuroplasticity. The effect of peripheral cortisol levels on neuroplastic changes involves brain regions that express glucocorticoid receptors, such as the hippocampus^5^. A recent study demonstrated that changes in peripheral cortisol levels under stress conditions have important implications on the functioning of neural structures of the default-mode network (DMN), including the hippocampus, which likely actively retrieves past experiences, anticipates future events^6^, and regulates our mind-wandering activity^7^. Furthermore, the intake of hydrocortisone may affect resting-state functional coupling of neural substrates that are important for emotional processing, such as the amygdala^8^. These findings suggest an important role of peripheral cortisol levels in cognitive and/or affective function and their association with neuroplastic changes.

More recently, mindfulness, which is defined as *“the awareness that emerges through paying attention on purpose, in the present moment, and nonjudgmentally to the unfolding of experience moment by moment*” 9, has been associated with cortisol levels^10^. In other words, increased awareness is implicated in mindfulness training. There have been reports showing that mindfulness training, e.g. in the form of meditation practice, modulates cortisol levels in healthy subjects^11^ and cancer patients^12^, plausibly via enhanced executive control promoted by nonjudgmental awareness of experiences in the present moment cultivated during the practice^13^. It is believed that the refined openness and awareness allow one to detect subtle changes in affective states with greater flexibility, which in turn reduces habitual tendencies of rumination and enhances adaptive emotion regulation^14^. However, the direct effect of training-induced changes in awareness on changes in baseline peripheral cortisol levels is controversial^15^,^16^,^17^.

There have been a surge of studies reporting the neuroplastic effects of meditation on the default mode network (DMN). Taylor *et al*.^18^ reported different functional connectivity in experienced meditators (with over 1000 hours of practice) relative to novices in DMN regions that are implicated in self-referential processing and emotional appraisal. Furthermore, Brewer *et al*.^19^ observed deactivation of the ventromedial prefrontal cortex and posterior cingulate cortex (PCC) and enhanced functional coupling between the PCC and the dorsal anterior cingulate cortex and dorsolateral prefrontal cortex, which are implicated in cognitive control and self-monitoring during resting states, in experienced meditators compared to novices. Yet, the effect of meditation training in beginning meditators is unknown. The current study examined the effect of changes of reported awareness induced by a short-term awareness-based compassion meditation (ABCM) on beginning meditators. To increase the overall range of changes in training-induced changes in awareness, we included a relaxation training group. The two trainings are expected to have similar effects on stress management and emotion regulation^20^,^21^. The main difference between the two trainings is expected to be on the cultivation of mindfulness associated with different levels of changes in awareness^22^. Awareness would increase with meditation training because meditation reinforced a focused state that enables people to sustain universal, non-referential love and compassion^23^. On the other hand, relaxation training does not emphasize the maintenance of awareness but the achievement of a relaxed state. We first explored whether mental training (ABCM/relaxation)-induced changes in cortisol levels correlated with changes in resting state activity using a whole-brain analysis. We hypothesized that training-induced changes in cortisol levels and resting state activity would be associated (which is referred to as ‘neural-cortisol association’ hereafter) in brain regions that are sensitive to cortisol, particularly the hippocampus and prefrontal cortex. On the other hand, the manifestation of mindfulness levels, and hence awareness, in different people is different^24^, and it is possible that these differences may affect the neural-cortisol association (if any). Therefore, our second aim was to examine in the whole brain whether the neural-cortisol association in the hippocampus and prefrontal cortex was moderated by the training-induced changes in awareness.

## Results

### Demographic data and plasma cortisol levels

Table 1 presents the demographic data and plasma cortisol levels at baseline and the changes in plasma cortisol levels after training. The two groups were matched in age [*t*(19) = −0.984, *p* = 0.337], gender composition [*X*^*2*^(1) = 0.398, *p* = 0.528] and years of education [*t*(19) = −1.949, *p* = 0.072]. The ABCM group had a lower body mass index (BMI) than the relaxation group [*t*(19) = −2.389, *p* = 0.027]. Since BMI has been reported to positively associate with plasma cortisol levels in healthy subjects^25^, BMI was included as a covariate in all regression models in this study. The drop-out rate did not differ between groups [*X*^*2*^(1) = 2.411, *p* = 0.121].

### Duration of practice

The two groups completed a similar total amount of home-based practice (ABCM: 509.1 ± 156.7 minutes; relaxation: 550.1 ± 244.4 minutes) [*t*(19) = −0.452, *p* = 0.656]. The average duration of practice of the ABCM group was 74.9 ± 26.5 minutes per week, and that of the relaxation group was 77.0 ± 33.1 minutes per week. There was no group difference in average duration of practice [*t*(19) = −0.164, *p* = 0.871)]. The average duration of practice of cultivation of attention and of practice of cultivation of compassion performed by the ABCM group was 416.8 (81.9% of total practice time) and 92.3 (18.1% of total practice time) minutes, respectively, based on the proportion of practice reported at post-training assessments. The average duration of practice on diaphragmatic breathing, progressive muscle relaxation, and imagery relaxation carried out by the relaxation group was 349.5 (64.1% of total practice time), 76.5 (13.9% of total practice time), and 124.1 (22.0% of total practice time) minutes, respectively.

### Self-reported mindfulness changes

Table 2 shows that both groups had similar mean values for the total score and all sub-scores on the Cognitive and Affective Mindfulness Scale Revised (CAMSR)^26^ at baseline. Group-by-Time ANOVA and ANCOVA (adjusted for BMI) demonstrated significant differences in changes in some mindfulness scores. Paired sample t-tests further confirmed that the ABCM group had a significant increase in the total score, and the relaxation group had a significant reduction in the awareness score.

### Association between awareness and cortisol changes

A significant negative correlation was found between changes in awareness and changes in cortisol levels across the groups (*β* = −0.562, *p* = 0.009, adjusted for BMI).

### Relationships between neural and cortisol changes

**Figure 1:**
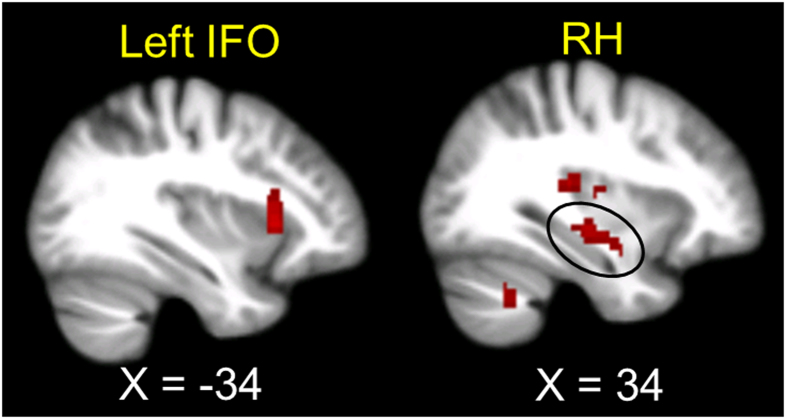
Significant negative association between concurrent neural changes and cortisol changes. Neural changes were measured with regional homogeneity (ReHo) during the resting state. For display purposes, an uncorrected voxel level of *p* < 0.005 was used. IFO = Anterior insula and adjacent frontal operculum; RH = Right hippocampus.

Changes in cortisol negatively correlated with changes in regional homogeneity (ReHo) in the right hippocampus (RH) and the left anterior insula and adjacent frontal operculum, which is referred to as the left IFO hereafter^27^ (Table 3 and Fig. 1). Although changes in cortisol also negatively correlated with changes in ReHo in the left hippocampus (LH), this effect was small (cluster = 7 voxels) and thus considered as subthreshold.

### Moderation effect of mindfulness on the neural-cortisol association

**Figure 2:**
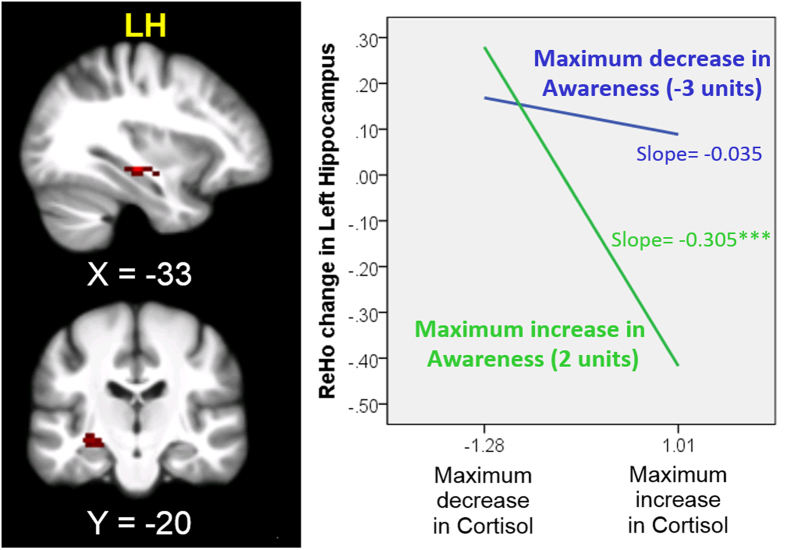
Moderation effect of awareness changes on neural–cortisol changes. Neural changes were measured with regional homogeneity (ReHo) during the resting state. The simple effects of cortisol within the maximum increase or decrease in awareness were estimated according to the procedures described by Aiken and West^49^; Jaccard, Turrisi and Wan^50^. The simple effect of cortisol within the maximum increase in awareness (slope = −0.305) was significant (*t* = −6.435, ***p < 0.00001), but not for the maximum decrease in awareness (slope = −0.035, *t* = −1.447, *p* = 0.167). For display purposes, an uncorrected voxel level of *p* < 0.005 was used. LH = Left hippocampus.

Changes in awareness significantly moderated the negative relationship between changes in cortisol levels and the ReHo of the LH. Taken the subthreshold negative correlation between changes in cortisol and ReHo of the LH into consideration, our findings imply that changes in ReHo of the LH are specifically associated with the interaction between changes in awareness and cortisol. Increases in awareness and decreases in cortisol levels were associated with increases in the ReHo of the LH (corrected *p* < 0.05, Table 4 and Fig. 2). Changes in cortisol (*t* = −6.717, *p* = 0.0000049), awareness (*t* = −3.370, *p* = 0.004) and the interaction of the two variables (*t* = −3.119, *p* = 0.007) were significant predictors of changes in the ReHo of the LH in a linear regression model (adjusted for BMI). The three variables accounted for 73% of the variance (adjusted R^2^) in changes in the ReHo of the LH. The standardized regression equation is as follows:


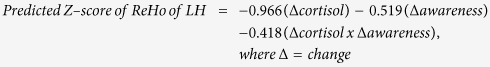


## Discussion

The current study applied both ABCM and relaxation training to induce a range of changes in awareness in our cohort. Our findings emphasize the moderating role of awareness on the neural-cortisol association. This is the first study that reports the moderation effect of training-induced changes in awareness on the relationship between concurrent neural (resting-state) changes and cortisol changes in healthy human subjects. There are three key observations. First, we revealed a novel negative correlation between cortisol levels and awareness. Second, we clearly demonstrated a longitudinal association of concurrent neural changes and cortisol changes in the ReHo of the RH and the left IFO during resting state. Third, increases and decreases in awareness had different effects on the association between cortisol levels and the ReHo of the LH during resting state. The regression model of changes in cortisol levels, awareness and their interaction collectively explained a large proportion of the variance in ReHo changes in the LH, in which changes in cortisol levels carried more weight than awareness and the interaction effect in the prediction of ReHo changes in the LH. Although the absolute change in cortisol levels observed in this study was within the normal range and small, these findings corroborate previous observations of the strong effect of cortisol and demonstrate a novel cortisol–mindfulness interaction on hippocampal activity, which suggests the importance of targeting peripheral cortisol and its interaction with mindfulness to maximize the efficacy of the design of interventions for people with hippocampal abnormalities. Overall, our findings supported our hypothesis that training-induced changes in awareness is a significant moderator in the relationship between concurrent neural changes and cortisol changes in the hippocampus. This study furthers our understanding of the inter-relationship between manipulation of the level of reported awareness and neural–cortisol interactions, and has implications for the behavioral treatment of patients with abnormal hippocampal activity during resting states.

The hippocampus is an area that regulates HPA function^28^. Associations between circulating cortisol levels and hippocampal changes have been reported. For example, morning serum cortisol levels are associated with regionally specific variations in hippocampal morphology during early development in healthy preadolescent children^29^. A more recent study has shown that saliva cortisol levels are negatively associated with hippocampal volume in young and older adults under stressful conditions^30^. However, little is known about the relationship between plasma cortisol level changes and the resting state of the hippocampus. Our findings showed a significant negative correlation between changes in plasma cortisol levels and the ReHo of the RH in our study cohort. These results support our hypothesis that concurrent neural (resting-state) changes and cortisol changes are detectable in cortisol-sensitive brain regions.

Similarly, a negative correlation with cortisol change was also found in the left IFO, which is implicated in processes relating to risk perception and salience evaluation. The anterior insula was activated consistently for decision and anticipation risks, particularly when potential losses were encountered^31^. One recent study found that the short-range functional connectivity dimension (which is similar to ReHo because it reflects correlations between one voxel and other voxels within a local cluster) in the bilateral inferior frontal gyri and adjacent anterior insula negatively correlated with general risk propensity in men and women^32^. The ReHo–cortisol association in the left IFO may have implications for psychiatric conditions that involve abnormal neural processes related to risk perception and risk-taking behavior, such as in drug abusers^33^, who display abnormal salivary cortisol levels^34^.

We observed a novel negative association between changes in plasma cortisol levels and awareness. This link may bridge other findings that relate mindfulness to cognitive-affective functions. Enhanced awareness of emotional experience or mental processes may reduce emotional arousal^35^, and mindfulness may counteract cognitive distortion and ruminative thinking^36^. We speculated that changes in plasma cortisol levels may mediate the effect of enhanced awareness of one’s own experience on reducing cognitive distortion and emotional arousal. However, this speculation requires further investigation.

We further examined the role of awareness in moderating ReHo–cortisol changes using a linear regression model that incorporated the interaction term of awareness and cortisol changes. We observed significant main effects of cortisol and awareness changes and interaction effects on the ReHo changes of LH, in which the three variables together predicted a large proportion of the variance in the changes in the ReHo of the LH. This finding indicates that plasma cortisol levels, awareness and their interaction are important factors that regulate neural activity in the LH. Comparisons of their standardized coefficients revealed that plasma cortisol levels were the most important of the three factors, which echoes the importance of cortisol homeostasis in neural function. Furthermore, the main effect of awareness changes on the ReHo of LH changes suggests that the effect of mindfulness meditation on neural activity changes may occur via behavioral changes in awareness. These observations corroborate a recent finding that also showed that mindfulness trait moderates a relationship between cortisol awakening response and psychological stress in healthy human subjects^24^. In that study, the cortisol awakening response was linked to psychological stress only in people with a low level dispositional mindfulness. In other words, a higher level of mindfulness may buffer the impact of stress on physiological arousal. The authors suggested that the tendency to be aware of experiences had beneficial effects on psychological and physiological well-being. In our samples, the ReHo of LH in subjects with an increase in awareness was more sensitive to peripheral cortisol changes (i.e. with a steeper slope). In other words, an increase in awareness was associated with a decrease in peripheral cortisol levels and an increase in resting-state synchrony in LH. These findings suggest that interventions focus only on reduction of peripheral cortisol levels may not be sufficient to restore a normal level of ReHo activity in LH, which was found to be relatively low in people with suicide attempts^37^ or those with higher trait anxiety^38^. On the other hand, increased ReHo of LH has been associated with better cognitive function^39^, implying interventions that could enhance awareness and reduce cortisol levels at the same time might be useful for people with mild cognitive impairment who might be capable of practicing meditation. This requires future research in a randomized controlled trial setting with larger sample size to confirm. Nevertheless, our findings suggest the moderation effect of awareness on the relationship between cortisol and resting state activity of the LH plays an important role in cognitive function in healthy subjects. Taken together, our findings support an increase in awareness, plausibly be induced by mindfulness training, could be useful in restoring the low ReHo of LH, together with reduction in peripheral cortisol levels. These findings might be beneficial to clinical populations such as patients with mild cognitive impairment (MCI), autism, major depressive disorder and Parkinson’s disease^3^,^40^,^41^,^42^. Future researches should focus on the effectiveness and dosage of treatment and predisposing factors, such as developmental stage, drug treatments and disease severity that might interfere with the treatment outcomes in the clinical population mentioned above.

This report is the first study to demonstrate the effect of training-induced changes in awareness on concurrent changes in the ReHo of the LH and the plasma cortisol level. We emphasized the influence of behavioral changes (e.g., awareness) in the monitoring of neural activity and its involvement in cortisol homeostasis. These findings pave the way for a more complete understanding of the inter-relationships between behavioral, endocrine and neural changes. The current findings may provide insight into new intervention approaches for age-related clinical conditions, such as MCI and Alzheimer’s disease, which are closely linked to a dysregulation of cortisol levels and hippocampal functions.

This study has several limitations. First, the sample size was relatively small; hence, the power of this study was limited. Furthermore, the small sample size did not allow conducting mediation analyses to deduce the relationship among changes in ReHo in the LH, changes in awareness and cortisol. The significant findings reported herein were rather robust, but future longitudinal studies should consider a larger sample to determine whether other significant differences will arise with the increased power. Second, our interpretations are limited by the study design. Subject bias cannot be totally eliminated because we adopted a quasi-experimental approach. For instance, the baseline between-groups difference in awareness score, though insignificant, might have an influence on the changes in awareness between the two groups. Also, participants who were less available might be busier or more employed and thus less likely to have completed the assessment earlier and was assigned to the relaxation group. However, some procedures were undertaken to minimize self-selection bias. Our data showed that both groups had similar drop-out rates and duration of practice, which suggests that they had similar levels of motivation and effort. Third, we did not include a passive control group in our study because of time and infrastructural constraints. Therefore, we cannot conclude that the observed changes are purely experimental effects rather than a maturation effect that occurs naturally with the passage of time. Fourth, our interpretation on the data could be limited to people with high motivation to both trainings. Also, exclusion of subjects who had a low participation rate could affect the generalizability of the current findings. Lastly, changes in awareness were assessed by only one self-reported questionnaire, which may limit the interpretation of our findings. Future studies should incorporate objective behavioural measures to confirm our findings, and adopt a randomization approach using active and passive control groups to further investigate these questions.

## Methods

### Participants

The Institutional Review Board of The University of Hong Kong (HKU) and the Hospital Authority approved this study; and the methods were carried out in accordance with the approved guidelines. A total of 27 right-handed Chinese participants were recruited from the local community and the alumni network of HKU via online flyers and email announcements. All participants gave their informed consent for participation. Exclusion criteria were magnetic resonance imaging (MRI) incompatibility, prior experience of meditation/relaxation training, history of brain injuries, neurological or psychiatric disease, and current engagement in any psychotherapy or pharmacotherapy that may affect the functioning of autonomic and/or central nervous systems. We also excluded participants who missed more than three out of seven training sessions. Inclusion criteria were 25–55 years old, with an interest in practicing both meditation and relaxation, and commitment to attending all lessons and assessments. Among the 27 enrolled participants, 15 of them were assigned to the ABCM group and the other 12 of them were assigned to the Relaxation Training group.

### Experimental procedures

A quasi-experimental design was adopted because of time and resource constraints (i.e., limited MRI scanning slots within three weeks before the start of ABCM or relaxation training). Since most participants applied for leave from work for assessments during daytime on weekdays, this may have minimized (if not totally eliminated) the possibility of job-related bias. Since participants were recruited and tested sequentially, and the ABCM training was launched first, the allocation of participants to training groups depended on when their pre-training assessments were completed. Those who completed the assessment before the start of ABCM training were assigned to the ABCM group. However, this underlying mechanism was unbeknown to participants, who were informed that they would be randomly assigned to either group. To ensure that they were equally motivated for both trainings, their group membership was announced after completing pre-training assessment and none dropped out because of the group assignment. Pre-training and post-training assessments were performed within 3 weeks of starting or finishing training. Both assessments included the same set of self-report questionnaires, brain scans, and blood collection. During the resting-state scan, participants were instructed to remain awake and fixate on a white crosshair on a dark screen for five minutes without focusing on any particular thoughts. Blood collection was conducted from 8–11a.m. to control for the effects of circadian fluctuations on cortisol levels^43^. On average, the pre- and post- blood taking time was 10:12am (standard deviation/SD = 40.5 minutes) and 10:14am (SD = 35.0 minutes) for the ABCM group. The pre- and post- blood taking time was 9:41am (SD = 26.1 minutes) and 9:31am (SD = 20.6 minutes) for the relaxation group. Participants who successfully completed the entire training and both assessments were compensated for their time and travelling expenses.

#### ABCM training

The meditation training taught basic techniques to enhance attention by listening closely to the sounds in the environment, one’s own breathing, and one’s own body condition, while cultivating compassion and kindness (*mettā*) toward the self and spreading it to people close to oneself, such as family members or friends, and finally to all living beings worldwide. The aim was to achieve mindfulness, peace, calm, and liberation. Each class started with 30–40 minutes of meditation practice followed by didactic teaching and discussion. The class ended with a short meditation practice. Participants attended two 1.5-hour lessons in the late evening for the first three weeks. They then engaged in self-practice for the subsequent three weeks. An intensive 2.5-hour group meditation session was held in the 5^th^ week. The meditation teacher was an experienced meditator with 12 years of meditation experience in the *Theravada* tradition who has taught meditation for two years.

#### Relaxation training

The class structure for relaxation training closely followed the meditation training. The relaxation techniques included diaphragmatic breathing, progressive muscle relaxation, and imagery relaxation. A short neuroscience lecture was given to match the didactic teaching in the meditation class. The relaxation teacher was a registered clinical psychologist who has taught relaxation to normal and clinical populations for two years.

### Amount of practice

Participants were given logbooks at the beginning of the training to record their daily practice in minutes. Participants were asked to reflect on the percentage of time they spent on different kinds of practice at the post-training assessment (e.g., cultivation of attention and compassion for the ABCM group). The amount of a specific practice (i.e. cultivation of attention and cultivation of compassion for ABCM training; diaphragmatic breathing, progressive muscle relaxation and imagery relaxation for relaxation training) performed by each participant was calculated by multiplying the percentage of time (%) that he/she spent on the specific practice with the total amount of practice (minutes) summarized across all logbooks.

### Self-reported mindfulness measure

The 10-item version of Cognitive and Affective Mindfulness Scale Revised (CAMSR)^26^ has 10 questions that are answered on a 5-point Likert scale. It has four sub-scores, namely the attention, present focus, awareness, and acceptance scores that measure the ability to regulate attention, orientation to present or immediate experience, awareness of experience, and attitude of acceptance or non-judgment towards experience, respectively. All sub-scales had three items except present focus had one item only. Higher CAMSR scores indicate more mindfulness. The reliability scores (Cronbach’s alpha) of the overall scale (10 items) and attention, awareness and acceptance sub-scales were 0.875, 0.781, 0.730 and 0.876, respectively. The Cronbach’s alpha of present focus was not available as there was only one item for this sub-scale in this study.

### Plasma cortisol levels

A registered nurse collected peripheral blood (3 ml) from each participant into EDTA tubes. Blood samples were immediately layered on Ficoll-Paque PLUS reagent from GE Healthcare Bio-Sciences (Pittsburgh, PA, USA, 1:1 v/v), and centrifuged at 400 × g for 40 min at room temperature. The upper plasma layer was collected and stored at −80 °C until further analysis. Plasma cortisol levels were measured using a commercially available ELISA kit from Quansys Biosciences (Logan, UT, USA) according to the manufacturer’s instructions. The detection range of the kit was 1.06–775 ng/ml. The intra-assay coefficient of variation (CV) of the ELISA kit was 6.5%, calculated from the duplicate of 20 samples, which is within the acceptable range^44^. The inter-assay CV is not applicable in this case, since only one ELISA plate was used in this study.

### Image acquisition

Whole-brain functional and anatomical scanning was performed using a 3.0 Tesla Philips Medical Systems Achieva scanner (see Supplementary Methods).

### Data analysis

Missing data was handled by mean substitution independently from each group. Overall, there were two subjects who were unable to provide blood sample in the pre- and post-assessments. Normality of any continuous data was examined using the Kolmogorov-Smirnov test. Non-normally distributed data were transformed using natural log transformation before any parametric analyses. The natural log transformed plasma cortisol levels from 21 participants were used in all analyses reported in this study. Between-group differences in demographic data were examined using the independent-samples *t*-test or *X*^*2*^(Chi-square) test, where appropriate. Between-group differences in baseline value were examined using independent-samples *t*-test and ANCOVA (adjusted for BMI). Group-by-time changes were examined using ANOVA and ANCOVA (adjusted for BMI). For any significant group-by-time change, a post-hoc two-tailed paired *t*-test was conducted. A *p*-value less than 0.05 was considered statistically significant. The association between changes in awareness and cortisol levels was examined by a linear regression model (adjusted for BMI) using SPSS.

Resting-state activity was examined using regional homogeneity (ReHo) in SPM8 and DPARSFA in REST (version1.8)^45^. ReHo is a widely used and reliable measure that represents local synchronization of spontaneous brain activities^46^. The first 10 volumes of data were discarded to ensure signal stability. The general preprocessing steps included slice-timing correction, realignment, co-registration, normalization, linear trend removal, and band-pass filtering at 0.01–0.08 Hz. Kendall’s coefficient of concordance (KCC)^47^ for each voxel was calculated Smoothing using a 6-mm full-width half-maximum (FWHM) Gaussian kernel was performed on standardized ReHo maps.

Difference maps for neural changes were generated by subtracting pre- from post-standardized ReHo maps. The change in cortisol was used as a predictor in a regression model to identify the brain regions where ReHo changes could be predicted by cortisol changes. Significance was defined as whole-brain corrected *p* < 0.05, determined by Monte Carlo simulations in AlphaSim (voxel-level uncorrected *p* < 0.001, k > 13 voxels, FWHM = 6 mm, rmm = 5, 1000 iterations). The resultant average ReHo values were extracted using REX as dependent variables for analyzing the strength of association with cortisol changes by linear regression models (adjusted for BMI) in SPSS.

Centered changes in awareness and cortisol (main effects), the product of these two changes (interaction term) and BMI (covariate) were used as continuous predictors in a regression model with data collapsed across treatment groups to examine whether changes in awareness moderated the significant association between ReHo-cortisol changes. Average ReHo values were extracted using REX for significant moderation effects. The entire regression model with the same continuous predictors was reconstructed in SPSS to calculate the adjusted R^2^ and standardized beta values of each predictor using multiple linear regression. The procedures suggested by Warner^48^ were adopted to graph the moderation effect of two quantitative variables (see Supplementary Methods).

## Additional Information

**How to cite this article**: Lau, W. K.W. *et al*. Can the neural-cortisol association be moderated by experience-induced changes in awareness? *Sci. Rep*. **5**, 16620; doi: 10.1038/srep16620 (2015).

## Supplementary Material

Supplementary Information

## Figures and Tables

**Table 1:** Descriptive statistics of demographic data and plasma cortisol levels at baseline and their change after training.

	ABCM (N = 10)	Relaxation(N = 11)	*t- or X*^*2*^ value	*p*-value		
Demographic data						
Age	37.8 ± 11.2	42.0 ± 8.1	−0.984	0.337		
Gender (Male: Female)	5: 5	4: 7	0.398	0.528		
Years of education	15.6 ± 5.4	19.4 ± 3.1	−1.949	0.072		
BMI	20.6 ± 2.4	23.6 ± 3.3	−2.389	**0.027**		
	**ABCM (N = 10)**	**Relaxation (N = 11)**	***t- or F-*****value**	***p*****-value**	**Adjusted for BMI**	
					***F-*****value**	***p*****-value**
Plasma cortisol levels (ng/ml)						
Baseline	82.6 ± 77.6	80.8 ± 105.0	*t* = 1.014	0.325	0.076	0.786
Change	−21.7 ± 78.4	−8.1 ± 91.3	*F* = 0.262	0.615	0.016	0.902

The values in each cell (except for the row of Gender) represent mean ± standard deviation. Between-groups difference in demographic data was determined by independent-samples *t*-tests or *X*^*2*^ (Chi-square) test (two-tailed), where appropriate. Baseline between-groups difference in natural log transformed cortisol levels was examined by two-tailed independent samples t-test (without any adjustment) and one-way ANCOVA (adjusted for BMI). Group-by-time change in natural log transformed cortisol levels was examined by two-way ANOVA (without any adjustment) and ANCOVA (adjusted for BMI). ABCM: Awareness-based compassion meditation; BMI: Body mass index.

**Table 2:**
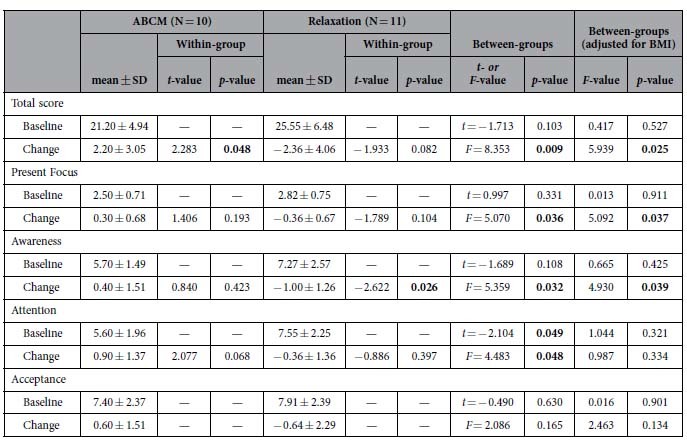
Mindfulness scores at baseline and their changes after training.

Within-group difference was examined by paired-sample *t*-test. Between-groups difference in baseline values was examined by two-tailed independent samples *t*-test (without any adjustment) and one-way ANCOVA (adjusted for BMI). Group-by-time change was examined by two-way ANOVA (without any adjustment) and ANCOVA (adjusted for BMI). ABCM: Awareness-based compassion meditation; BMI: Body mass index; CAMSR: Cognitive and affective mindfulness scale revised; SD: Standard deviation.

**Table 3:**
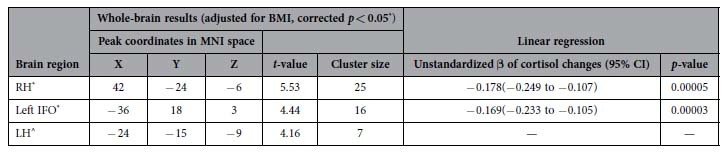
Negative association between changes in ReHo and cortisol changes.

Unstandardized β of cortisol changes was calculated from linear regression of ReHo changes predicted by natural log transformed cortisol changes, adjusted for body-mass index (BMI). CI: Confidence interval; IFO: Anterior insula and adjacent frontal operculum; LH: Left Hippocampus; MNI: Montreal Neurological Institute; ReHo: Regional homogeneity; RH: Right hippocampus.

^*^Whole-brain corrected *p* < 0.05, determined by AlphaSim (voxel-level uncorrected *p* < 0.001, k > 13 voxels).

^^^A subthreshold effect was observed on the LH, of which the ReHo changes also negatively associated with cortisol changes. This suggested that the association between changes in ReHo of the LH and changes in cortisol alone was weak.

**Table 4:** Moderation effect of awareness changes on the negative association between changes in ReHo and cortisol changes (adjusted for body mass index).

Brain region	Peak coordinates in MNI space			*F-*value	Cluster size
	X	Y	Z		
LH	−36	−24	−9	33.01	39

Note: LH: Left hippocampus; MNI: Montreal Neurological Institute; ReHo: Regional homogeneity.

## References

[b1] Daskalakis N. P., Bagot R. C., Parker K. J., Vinkers C. H. & de Kloet E. R. The three-hit concept of vulnerability and resilience: toward understanding adaptation to early-life adversity outcome. Psychoneuroendocrinology 38, 1858–1873 (2013). 23838101 10.1016/j.psyneuen.2013.06.008 PMC3773020

[b2] McEwen B. S. The ever-changing brain: cellular and molecular mechanisms for the effects of stressful experiences. Dev. Neurobiol. 72, 878–890 (2012). 21898852 10.1002/dneu.20968 PMC3248634

[b3] Cubala W. J. & Landowski J. C-reactive protein and cortisol in drug-naive patients with short-illness-duration first episode major depressive disorder: possible role of cortisol immunomodulatory action at early stage of the disease. J. Affect. Disord. 152–154, 534–537 (2014). 10.1016/j.jad.2013.10.004 24161452

[b4] Horn C. A., Pietrzak R. H., Corsi-Travali S. & Neumeister A. Linking plasma cortisol levels to phenotypic heterogeneity of posttraumatic stress symptomatology. Psychoneuroendocrinology 39, 88–93 (2014). 24275007 10.1016/j.psyneuen.2013.10.003 PMC3843152

[b5] Montaron M. F. . Lifelong corticosterone level determines age-related decline in neurogenesis and memory. Neurobiol. Aging 27, 645–654 (2006). 15953661 10.1016/j.neurobiolaging.2005.02.014

[b6] Vaisvaser S. . Neural traces of stress: cortisol related sustained enhancement of amygdala-hippocampal functional connectivity. Front. Hum. Neurosci. 7, 313 (2013). 23847492 10.3389/fnhum.2013.00313 PMC3701866

[b7] Raichle M. E. . A default mode of brain function. Proc. Natl. Acad. Sci. USA 98, 676–682 (2001). 11209064 10.1073/pnas.98.2.676 PMC14647

[b8] Henckens M. J., van Wingen G. A., Joels M. & Fernandez G. Corticosteroid induced decoupling of the amygdala in men. Cereb. Cortex 22, 2336–2345 (2012). 22079927 10.1093/cercor/bhr313

[b9] Kabat-Zinn J. Mindfulness-Based Interventions in Context: Past, Present, and Future. Clin. Psychol. Sci. Pract. 10, 144–156 (2003).

[b10] Jacobs T. L. . Self-reported mindfulness and cortisol during a Shamatha meditation retreat. Health Psychol. 32, 1104–1109 (2013). 23527522 10.1037/a0031362

[b11] Rosenkranz M. A. . A comparison of mindfulness-based stress reduction and an active control in modulation of neurogenic inflammation. Brain. Behav. Immun. 27, 174–184 (2013). 23092711 10.1016/j.bbi.2012.10.013 PMC3518553

[b12] Branstrom R., Kvillemo P. & Akerstedt T. Effects of mindfulness training on levels of cortisol in cancer patients. Psychosomatics 54, 158–164 (2013). 23218058 10.1016/j.psym.2012.04.007

[b13] Hölzel B. K. . How does mindfulness meditation work? Proposing mechanisms of action from a conceptual and neural perspective. Perspect. Psychol. Sci. 6, 537–559 (2011). 26168376 10.1177/1745691611419671

[b14] Teper R., Segal Z. V. & Inzlicht M. Inside the mindful mind: how mindfulness enhances emotion regulation through improvements in executive control. Curr. Dir. Psychol. Sci. 22, 449–454 (2013).

[b15] Lipschitz D. L., Kuhn R., Kinney A. Y., Donaldson G. W. & Nakamura Y. Reduction in salivary alpha-amylase levels following a mind-body intervention in cancer survivors–an exploratory study. Psychoneuroendocrinology 38, 1521–1531 (2013). 23375640 10.1016/j.psyneuen.2012.12.021 PMC3686861

[b16] Stefanaki C. . Impact of a mindfulness stress management program on stress, anxiety, depression and quality of life in women with polycystic ovary syndrome: a randomized controlled trial. Stress 18, 57–66 (2015). 25287137 10.3109/10253890.2014.974030

[b17] Turakitwanakan W., Mekseepralard C. & Busarakumtragul P. Effects of mindfulness meditation on serum cortisol of medical students. J. Med. Assoc. Thai. 96 Suppl 1, S90–95 (2013). 23724462

[b18] Taylor V. A. . Impact of meditation training on the default mode network during a restful state. Soci. Cogn. Affect. Neurosci. 8, 4–14 (2013). 10.1093/scan/nsr087 PMC3541485 22446298

[b19] Brewer J. A. . Meditation experience is associated with differences in default mode network activity and connectivity. Proc. Natl. Acad. Sci. USA 108, 20254–20259 (2011). 22114193 10.1073/pnas.1112029108 PMC3250176

[b20] Alexopoulos E. C., Zisi M., Manola G. & Darviri C. Short-term effects of a randomized controlled worksite relaxation intervention in Greece. Ann. Agric. Environ. Med. 21, 382–387 (2014). 24959794 10.5604/1232-1966.1108609

[b21] Onieva-Zafra M. D., García L. H. & Del Valle M. G. Effectiveness of guided imagery relaxation on levels of pain and depression in patients diagnosed with fibromyalgia. Holist. Nurs. Pract. 29, 13–21 (2015). 25470476 10.1097/HNP.0000000000000062

[b22] Feldman G., Greeson J. & Senville J. Differential effects of mindful breathing, progressive muscle relaxation, and loving-kindness meditation on decentering and negative reactions to repetitive thoughts. Behav. Res. Ther. 48, 1002–1011 (2010). 20633873 10.1016/j.brat.2010.06.006 PMC2932656

[b23] Salzberg S. Loving-kindness: The revolutionary art of happiness. (Boston: Shambhala, 1995).

[b24] Daubenmier J., Hayden D., Chang V. & Epel E. It’s not what you think, it’s how you relate to it: dispositional mindfulness moderates the relationship between psychological distress and the cortisol awakening response. Psychoneuroendocrinology 48, 11–18 (2014). 24971591 10.1016/j.psyneuen.2014.05.012 PMC4503930

[b25] Syed S. B. & Qureshi M. A. Association of aldosterone and cortisol with cardiovascular risk factors in prehypertension stage. Int. J. Hypertens 2012, ID906327 (2012). 10.1155/2012/906327 PMC3431092 22957211

[b26] Feldman G., Hayes A., Kumar S., Greeson J. & Laurenceau J. P. Mindfulness and emotion regulation: the development and initial validation of the cognitive and affective mindfulness scale-revised (CAMS-R). J. Psychopathol. Behav. Assess. 29, 177–190 (2007).

[b27] Jabbi M., Swart M. & Keysers C. Empathy for positive and negative emotions in the gustatory cortex. Neuroimage 34, 1744–1753 (2007). 17175173 10.1016/j.neuroimage.2006.10.032

[b28] Rubin R. T., Mandell A. J. & Crandall P. H. Corticosteroid responses to limbic stimulation in man: localization of stimulus sites. Science 153, 767–768 (1966). 5940897 10.1126/science.153.3737.767

[b29] Wiedenmayer C. P. . Cortisol levels and hippocampus volumes in healthy preadolescent children. Biol. Psychiatry 60, 856–861 (2006). 16603131 10.1016/j.biopsych.2006.02.011 PMC2367228

[b30] Sindi S. . Now you see it, now you don’t: testing environments modulate the association between hippocampal volume and cortisol levels in young and older adults. Hippocampus 24, 1623–1632 (2014). 25112535 10.1002/hipo.22341

[b31] Mohr P. N., Biele G. & Heekeren H. R. Neural processing of risk. J. Neurosci. 30, 6613–6619 (2010). 20463224 10.1523/JNEUROSCI.0003-10.2010 PMC6632558

[b32] Zhou Y. . The neural correlates of risk propensity in males and females using resting-state fMRI. Front. Behav. Neurosci. 8, 2 (2014). 24478649 10.3389/fnbeh.2014.00002 PMC3904110

[b33] Stewart J. L., Juavinett A. L., May A. C., Davenport P. W. & Paulus M. P. Do you feel alright? Attenuated neural processing of aversive interoceptive stimuli in current stimulant users. Psychophysiology 52, 249–262 (2015). 25183168 10.1111/psyp.12303 PMC5962278

[b34] Jaremko K. M., Sterling R. C. & Van Bockstaele E. J. Psychological and physiological stress negatively impacts early engagement and retention of opioid-dependent individuals on methadone maintenance. J. Subst. Abuse Treat. 48, 117–127 (2015). 25239858 10.1016/j.jsat.2014.08.006 PMC4250337

[b35] Herwig U., Kaffenberger T., Jancke L. & Bruhl A. B. Self-related awareness and emotion regulation. Neuroimage 50, 734–741 (2010). 20045475 10.1016/j.neuroimage.2009.12.089

[b36] Sears S. & Kraus S. I think therefore I om: cognitive distortions and coping style as mediators for the effects of mindfulness meditation on anxiety, positive and negative affect, and hope. J. Clin. Psychol. 65, 561–573 (2009). 19241400 10.1002/jclp.20543

[b37] Cao J. . Abnormal regional homogeneity in young adult suicide attempters with no diagnosable psychiatric disorder: a resting state functional magnetic imaging study. Psychiatry Res. 231, 95–102 (2015). 25496980 10.1016/j.pscychresns.2014.10.011

[b38] Hahn T., Dresler T., Pyka M., Notebaert K. & Fallgatter A. J. Local synchronization of resting-state dynamics encodes Gray’s trait Anxiety. PLoS One 8, e58336 (2013). 23520499 10.1371/journal.pone.0058336 PMC3592924

[b39] Yu R. . Cognitive enhancement of healthy young adults with hyperbaric oxygen: a preliminary resting-state fMRI study. Clin. Neurophysiol. (2015), doi: 10.1016/j.clinph.2015.01.010. [Epub ahead of print]. 25703942

[b40] Tordjman S. . Altered circadian patterns of salivary cortisol in low-functioning children and adolescents with autism. Psychoneuroendocrinology 50, 227–245 (2014). 25244637 10.1016/j.psyneuen.2014.08.010

[b41] Wang Y. . Using regional homogeneity to reveal altered spontaneous activity in patients with mild cognitive impairment. Biomed. Res. Int. 2015, 807093 (2015). 25738156 10.1155/2015/807093 PMC4337114

[b42] Yang H. . Changes in spontaneous brain activity in early Parkinson’s disease. Neurosci. Lett. 549, 24–28 (2013). 23769726 10.1016/j.neulet.2013.05.080

[b43] Touitou Y. & Haus E. Alterations with aging of the endocrine and neuroendocrine circadian system in humans. Chronobiol. Int. 17, 369–390 (2000). 10841211 10.1081/cbi-100101052

[b44] Food and Drug Association, *Guidance for industry – bioanalytical method validation*. (2001) Available at: http://www.fda.gov/downloads/Drugs/GuidanceComplianceRegulatoryInformation/Guidances/UCM070107.pdf (Accessed: 12^th^ March 2015).

[b45] Song X. W. . REST: a toolkit for resting-state functional magnetic resonance imaging data processing. PLoS One 6, e25031 (2011). 21949842 10.1371/journal.pone.0025031 PMC3176805

[b46] Zang Y., Jiang T., Lu Y., He Y. & Tian L. Regional homogeneity approach to fMRI data analysis. Neuroimage 22, 394–400 (2004). 15110032 10.1016/j.neuroimage.2003.12.030

[b47] Kendall M. G. & Gibbons J. D. Rank correlation methods. 5th edn, (E. Arnold: Oxford University Press, 1990).

[b48] Warner R. M. Applied statistics: from bivariate through multivariate techniques. 2nd edn (SAGE Publications, 2013).

[b49] Aiken L. S. & West S. G. Multiple regression: testing and interpreting interactions. (SAGE Publications, 1991).

[b50] Jaccard J., Turrisi R. & Wan C. K. Interaction effects in multiple regression. (SAGE Publications, 1990). 10.1207/s15327906mbr2504_4 26820822

