# Supplementary material for: Can the neural–cortisol association be moderated by experience-induced changes in awareness?
Source: Sci Rep. 2015 Nov 18;5:16620. doi: 10.1038/srep16620 (PMC4649618; doi:10.1038/srep16620)
Supplement: Supplementary Information [file srep16620-s1.doc]

**Can the neural–cortisol association be moderated by experience-induced changes in awareness?**

Way K.W. Lau, Mei-Kei Leung, Chetwyn C.H. Chan, Samuel S.Y. Wong, Tatia M.C. Lee

**Supplementary Methods**

**Methods**

***Participants***

The online flyers were posted on social websites and spread via social groups such as facebook and whatsapp groups. The email announcements were sent to the alumni of the University of Hong Kong (HKU) based on the alumni mailing list with the help of alumni association of the HKU.

***Image acquisition***

Whole-brain axial scanning was performed using a 3.0 Tesla Philips Medical Systems Achieva scanner equipped with an 8-channel SENSE head coil. Thirty-two functional slices were acquired using a T2*-weighted gradient echo planar imaging sequence [slice thickness=4 mm, time-to-repetition (TR)=1800 ms, time-to-echo (TE)=30 ms, flip angle=90°, matrix=64×64, field-of-view (FOV)=230×230×128 mm, voxel size=3.59×3.59×4 mm^3^]. The axial slices were adjusted to be parallel to the AC-PC plane. The first six volumes were discarded to allow for T1 equilibration effects. A three-dimensional, T1-weighted, magnetization-prepared rapid-acquisition gradient-echo (MP-RAGE) sequence was used to acquire high-resolution anatomical images [164 contiguous sagittal slices, 1-mm thick, TR=7 ms, TE=3.2 ms, flip angle=8°, FOV=164 mm, matrix=256x240 mm, voxel size=1 mm^3^].

***Data analysis***

In SPM, the neural-cortisol change correlation was done by setting up a contrast that tested if the correlation slope differs from zero positively (i.e. > 0) or negatively (i.e. < 0) using t-tests. Therefore, *t*-value was provided in Table 3. Since all analyses in SPM were thresholded at corrected *p*<0.05 (which is equal to a combined voxel-extent threshold of uncorrected *p*<0.001 and cluster size >13 as determined by AlphaSim), no individual corrected *p*-value was displayed for each brain cluster that has a significant correlation with cortisol changes in Table 3. Similarly, the moderation analysis in Table 4 was also thresholded at corrected *p*<0.05 in SPM.

The maximum increase and decrease of awareness and cortisol were selected as representative values for awareness and cortisol predictors, and these values were used to produce their corresponding interaction terms (for plotting the interactions only). The predicted values of outcome variables (ReHo) were generated using these predictors and interaction terms and their corresponding beta values. Using the predicted ReHo values and the selected awareness and cortisol predictors, the overall moderation effect was graphed.
